# Dataset of single and double faults scenarios using vibration signals from a rotary machine

**DOI:** 10.1016/j.dib.2023.109358

**Published:** 2023-07-04

**Authors:** Larry Marshall, David Jensen

**Affiliations:** University of Arkansas, Fayetteville, Arkansas 72701, USA

**Keywords:** Vibration, Bearings, Fault, Multiple faults

## Abstract

This dataset includes vibration sensor data from accelerometers located on the support bearings on a rotary machine designed as a fault simulator. Data collection for known faulty components include: bearing inner and outer raceway faults and bent shaft. 38 singles and double fault scenarios and a one no fault scenario were collected at three different operating frequencies (shaft rpm). Data was collected for approximately 10 seconds per scenario at a rate of 6400 hertz. Data can be used for machine learning classification.

**Specifications Table**

**Table:** 

Subject	Manufacturing Engineering
Specific subject area	Rotary Machine Vibrations
Type of data	Table
How the data were acquired	A fault simulator is composed of a motor spinning a shaft supported between two bearings. Additionally, a pulley and belt are attached to the shaft and are used to drive a gear box. A tachometer measured shaft rpm. Piezoelectric Accelerometers were attached in 3 perpendicular directions to the bearing housings and two directions on the gearbox housing. One or more faulty components were installed for each scenario. Signal data was acquired using a National Instruments Compact Data Acquisition system while running NI-DAQmx software.
Data format	Raw
Description of data collection	To gather data for a fault scenario, a nominal component on the machine fault simulator was replaced with a specified faulty component. In the case of two-fault scenarios, two nominal components were replaced with known specific faulty components. In the case of the nominal scenario, no components were replaced. The motor was turned on and set to one of three rpm values for each scenario. After reaching steady operating speed, data collection was conducted from sensors for 10 seconds. After data collection the machine simulator was turned off and the faulty components were either replaced with the nominal components or the next faulty component to be tested.
Data source location	*•* Institution: University of Arkansas • City/Town/Region: Fayetteville • Country: United States of Americ*a*
Data accessibility	Repository name: figshare.com Data identification number: DOI:https://doi.org/10.6084/m9.figshare.22693120.v1 Direct URL to data: https://figshare.com/articles/dataset/Single_and_Double_Fault_Scenarios_for_a_Rotary_Machine/22693120 Citation: [1] Jensen, David (2023). Single and Double Fault Scenarios for a Rotary Machine. figshare. Dataset. https://doi.org/10.6084/m9.figshare.22693120.v1

## Value of the Data

• These data are useful for exploring the challenges of classification of single and multiple faults in a rotary machines, which are a subsystem for many manufacturing systems*.*
• Researchers in the area of machine learning and condition-based maintenance can use these data for algorithm performance testing and research in fault classification and diagnosis.
• Comparison of these data to other fault-induced vibration data could provide a comparison across machines running similar experiments.

## 1 Objective

This dataset was created to train and test machine learning models that classify various single and multi-fault scenarios. The overall goal is to understand the challenges of classification in the context of single and multiple fault scenarios for machines [2].

Vibration data is commonly used to evaluate the health of rotary machine components [3]. Specifically, rolling element bearing faults, warped transmission shafts, and unbalanced shafts all induce vibration in a rotary machine. Vibration is measured through accelerometers mounted at locations on the machine. Both the type of component fault and the location of the fault affect the vibration signature. Additionally, the rotational speed of the machine also affects the vibration profile of the machine. The objective of the dataset described in this paper is to support research in the identification and classification of rotary machine faults.

Machine learning classification is one approach to implement machine health management. The goal of system health management is to enable condition-based maintenance, where parts are replaced as needed, as opposed to scheduled maintenance where parts may be replaced prior to failure and increase system down-time [4]. Machine learning (ML) classification algorithms can be used to identify faults based on experimental training data such as the dataset described in this work. It is an open research question how to develop and evaluate the accuracy classification for multiple fault scenarios.

One important aspect of training is developing and using feature extraction from raw data. Most ML classification algorithms use these features to group and match data rather than raw data. For this work, we have use signal characteristics such as mean, max, min, skewness, etc., as characteristic features in a similar approach as Gunerkar and Jalan in [5]. Another potential approach is to use frequency domain data for classification as demonstrated by Luwei et al. [6].

## 2 Data Description

Each CSV file in the dataset represents one scenario. This dataset includes 1 no-fault and 38 faulted scenarios for the machine operated at 3 different rpms for a total 114 csv files. At each operating rpm, the data set includes 11 single faults and 27 multi-fault scenarios.

All data were collected using a sampling rate of 6.4KHz for 10 seconds resulting in 64,000 data points for each scenario. Data for each scenario was collected 25 times for 3 different motor rpms. During these experiments, 8 piezoelectric accelerometers were located on the motor, on both bearing housings in x, y, and z directions, and on the gearbox.

All CSV files are formatted with sensor data in identical columns and named with the description of the fault location and type.

## 3 Experimental Design, Materials, and Methods

This experiment employed the Spectra Quest Machinery Fault Simulator in conjunction with a National Instruments Compact DAQ system and piezoelectric accelerometers with an accuracy of 102mg/V, and DAQmx software for data recording. The experiment was initiated by swapping components of the fault simulator in accordance with the desired fault scenario. Subsequently, the shaft frequency was established, and following its acceleration to the target frequency, the vibrations of each accelerometer were measured utilizing the Compact DAQ system and DAQmx software. These recordings were then saved as CSV files that can be used for further analysis.

### 3.1 Configuration

**Fig. 1:**
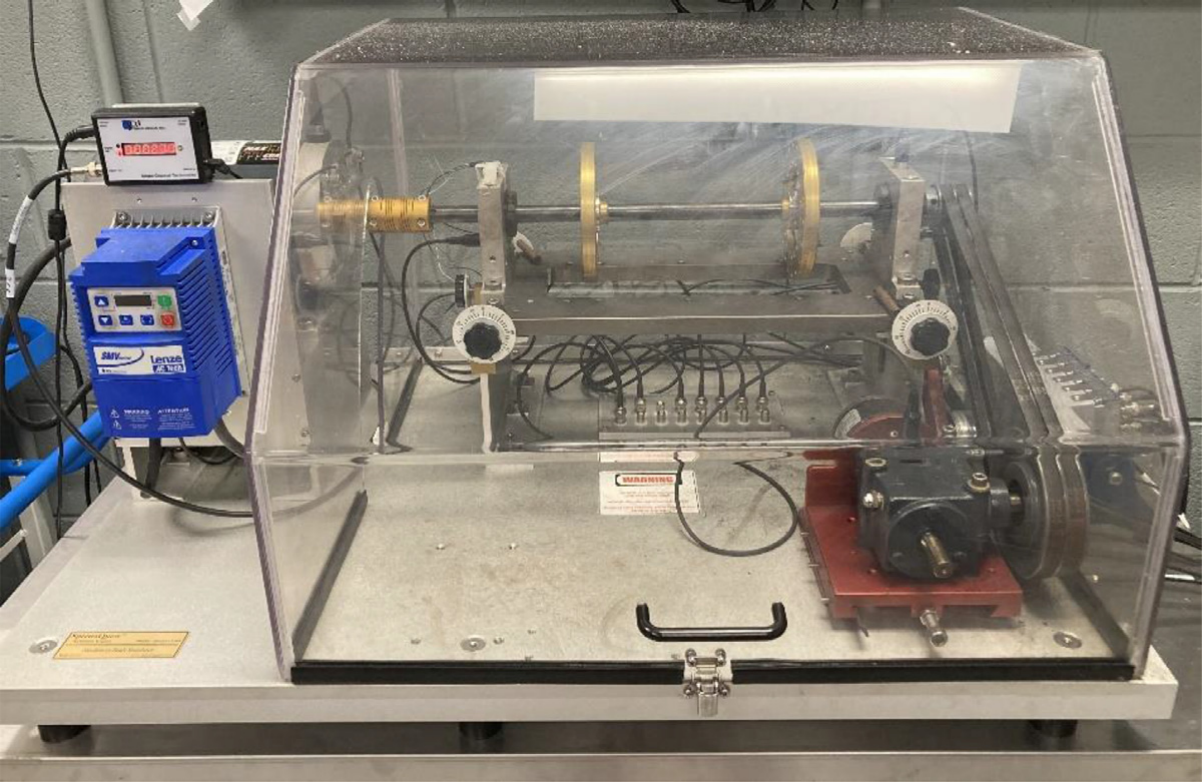
Rotary Machine Fault Simulator used for data collection.

The dataset described includes vibration data from multiple accelerometers mounted on a rotary machine fault simulator. This simulator is produced by SpectraQuest Inc. And shown in Fig. 1. From left to right, the setup includes a DC motor which drives a shaft supported by a left and right bearing. These are labeled in the data as Bearing 1 and Bearing 2 respectively. Between the bearings the shaft supports two weighted disks. Finally, to the right of bearing 2 a pulley and belt drive operate a gear box. This platform mimics many of the rotary components of various manufacturing machines.

### 3.2 Faulty components

**Fig. 2:**
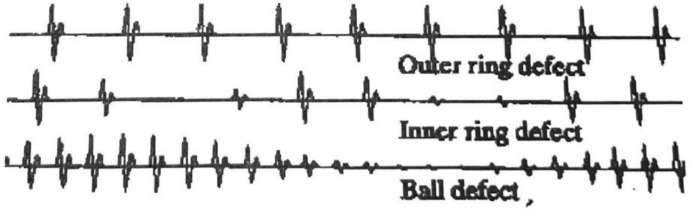
Example of expected time domain impact response of bearing defects.

One or more known faulty components is used to generate each scenario. The faults include four types of bearing faults and two types of shaft faults. The faulty bearings can be placed at either location, however, only one type of each fault was tested. That is, both bearings could not have the same faulty component used. The bearing faults where: 1) a defect in the inner raceway, 2) a defect in the outer raceway, 3) a defect on one of the ball elements, and 4) a combination of ball and raceway defect. These are known to produce different vibrational signals. Fig. 2 shows an example of the expected time-series data for these types of faults. Actual values depend on operating frequency and size of the defect. The two fault shafts are bent at prescribed locations. These are 1) centrally bent (between the two bearings), and 2) bent near the motor coupling.

**Fig. 3:**
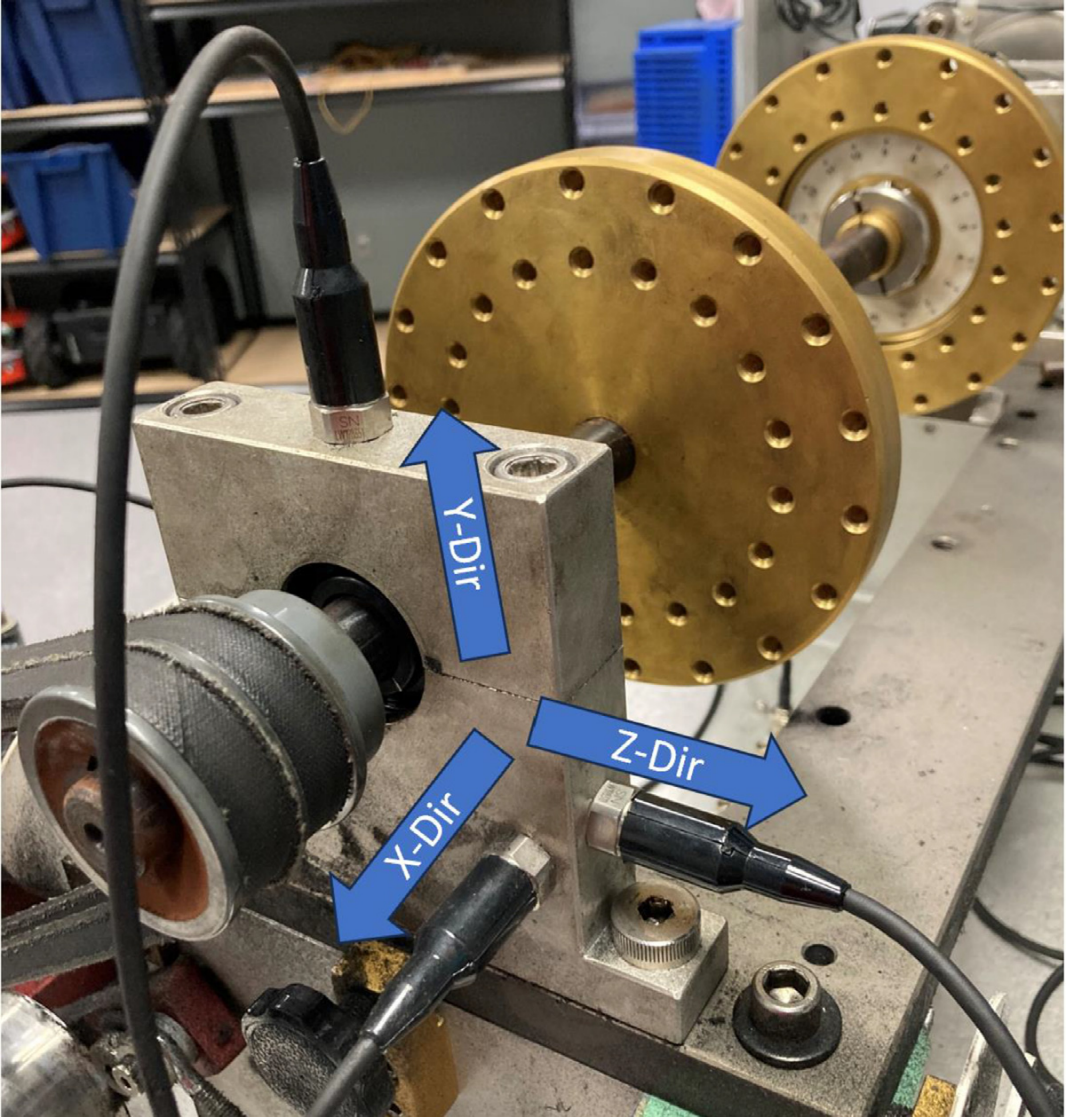
Accelerometers attached to the bearing housing in three labeled axial directions.

The data set includes multiple trials of scenarios for each individual fault mode as well as all the possible combinations of two faulty components. Noting the restriction mentioned above that a faulty component could not be used twice, and the system only contains one shaft component. The scenario data were collected using ICP accelerometers (model 608A11) [7] mounted on the bearing housings as shown in Fig. 3.

### 3.3 Procedure

Data were collected using the following procedure:

1. Turn on computer and data acquisition system.
2. Open DAQmx application.
3. Disassemble Spectra Quest fault simulator to access desired part.
4. Swap part with faulted part
5. Reassemble fault simulator ensuring to tighten bolts the same with each trial.
6. Attach accelerometers.
7. Check DAQmx software to see if all accelerometers are working properly.
8. Name trial scenario in DAQmx software
9. Set desired rpm on fault simulator.
10. Allow fault simulator to accelerate to the desired rpm.
11. In software specify recording time desired.
12. Record accelerations.
13. Export to CSV file.
14. Repeat steps 3-13 for each fault scenario.

## Ethics Statement

This work does not involve human subjects, animal experiments or any data associated with social networks.

## CRediT authorship contribution statement

**Larry Marshall:** Investigation, Writing – original draft. **David Jensen:** Writing – review & editing.

## Declaration of Competing Interests

X - The authors declare that they have no known competing financial interests or personal relationships that could have appeared to influence the work reported in this paper.

## Data Availability

Single and Double Fault Scenarios for a Rotary Machine (Original data) (Figshare.com).
